# Xanthoma of Bone: A Case Report

**DOI:** 10.1155/2012/986952

**Published:** 2012-04-08

**Authors:** Hasnae Guerrouj, Ayat Mouaden, Nouzha BenRais

**Affiliations:** Nuclear Medicine Department, University of Mohammed V-Souissi, Rabat, Morocco

## Abstract

Bone xanthoma is a rare disease due to the presence of cholesterol deposits in the bone. We report a case of a 56-year-old patient who was hospitalized in orthopedic department for fracture on the left humerus. Histological examination of bone biopsy performed at this site has objectified cholesterol deposits. Laboratory tests showed hypercholesterolemia with hypertriglyceridemia. The diagnosis of bone xanthoma was selected. The fracture was treated surgically. An initial bone scan revealed bone lesions localized especially at the humerus and femur. The patient was put under fenofibrate and statins. The evolution was marked by gradual regression of lesions in bone scans of control.

## 1. Introduction

Xanthoma is a benign lesion whose origin is abnormal deposition of cholesterol deposits on parts of the body other than blood vessels. This leads to an inflammatory cell infiltration. This lesion contains abundant foamy histiocytes most commonly occurring in superficial soft tissues such as skin, subcutis, or tendon sheaths. The involvement of deep skeletal structures, however, is rare. Most xanthomas occur in patients with hyperlipidemic disorders. We present a case of intraosseous xanthoma localized in long bones, review the literature, and demonstrate the value of bone scintigraphy in the management of this benign bone disease.

## 2. Observation

**Figure 1:**
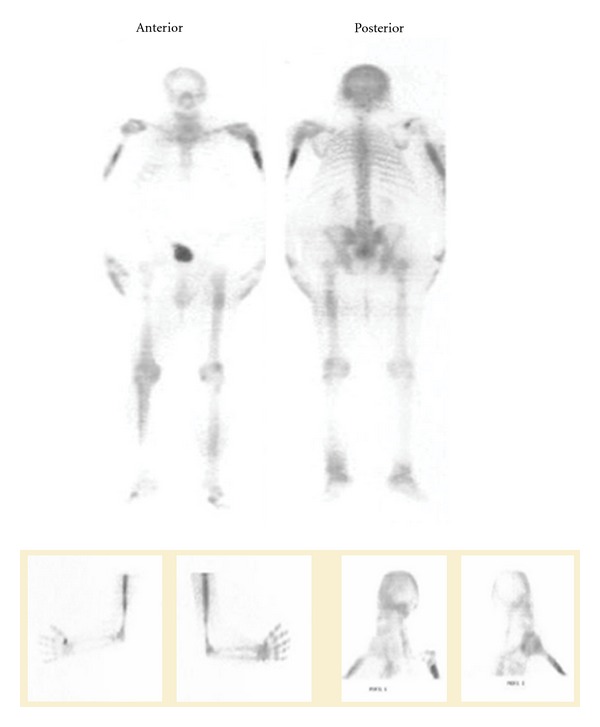
HMDP-Tc99m bone scan (anterior and posterior): increased uptake localised in diaphyses of humerus and femurs.

A 56-year-old male patient, without history of significant disease, has been hospitalized in the orthopedic department for fracture of the left humerus. X-ray showed clear images within bone. An initial HMDP-Tc99m bone scan revealed bone lesions localized especially at the humerus and femur (Figure 1). Histological examination of bone biopsy performed at the fracture site objectified cholesterol deposits. Laboratory tests showed hypercholesterolemia with hyper triglyceridemia. The diagnosis of bone xanthoma was selected. The fracture was treated surgically. The patient was put under 160 mg/jour of fenofibrate and 40 mg/jour of statin drug. The evolution was marked by gradual regression of lesions in bone scans of control. We did not notice any recurrence during four years of the followup.

## 3. Discussion

Bone xanthoma is a rare disorder due to the presence of cholesterol deposits in the bone. It is usually found in hyperlipidemia and hyperlipoproteinemia family. These are characterized by elevated levels of cholesterol that form deposits in soft tissue and bone [1, 2]. These are also reported in nonhyperlipidemic states [3, 4]. The most frequent location of bone xanthoma is the diaphysis of long bones, especially the tibia. Other locations may be particularly in the facial skeleton, mastoid air cells [5–7], and mandibular bone [8]. The skull can be affected (temporal [5–7] or frontal [9]) which may cause cerebellar compression.

The axial skeleton is not spared. A xanthoma case of sacrum [10] and calcaneus [11] has been described in the literature. Xanthoma of bone is characterized as a lytic lesion, often with cortical expansion or disruption [12]. X-ray images show intraosseous clear images sometimes resulting in deformities of the bone. Bone scintigraphy allows for mapping of lesions and posttherapy followup. It highlights increased uptake of long bones diaphyses, skull, and some bones of the face. The diagnosis is histological. Other diagnoses must be ruled out such as histiocytosis X, Erdheim-Chester, and clear cell carcinoma metastasis. The treatment is medical and surgical.

## 4. Conclusion

Bone xanthoma is a benign disease whose prognosis is good even after partial excision. The role of bone scintigraphy is useful in the staging of the disease and in therapeutic monitoring.
